# High Selectivity Hydrogen Gas Sensor Based on WO_3_/Pd-AlGaN/GaN HEMTs

**DOI:** 10.3390/s23073465

**Published:** 2023-03-26

**Authors:** Van Cuong Nguyen, Ho-Young Cha, Hyungtak Kim

**Affiliations:** School of Electronic and Electrical Engineering, Hongik University, Seoul 04066, Republic of Korea

**Keywords:** hydrogen sensor, gallium nitride, selectivity, palladium catalyst, tungsten trioxide, high electron mobility transistor

## Abstract

We investigated the hydrogen gas sensors based on AlGaN/GaN high electron mobility transistors (HEMTs) for high temperature sensing operation. The gate area of the sensor was functionalized using a 10 nm Pd catalyst layer for hydrogen gas sensing. A thin WO_3_ layer was deposited on top of the Pd layer to enhance the sensor selectivity toward hydrogen gas. At 200 °C, the sensor exhibited high sensitivity of 658% toward 4%-H_2_, while exhibiting only a little interaction with NO_2_, CH_4_, CO_2_, NH_3_, and H_2_S. From 150 °C to 250 °C, the 10 ppm hydrogen response of the sensor was at least eight times larger than other target gases. These results showed that this sensor is suitable for H_2_ detection in a complex gas environment at a high temperature.

## 1. Introduction

It is predicted that the Earth is facing a critical global warming situation. If the average temperature rises by 1.5 °C above pre-industrial levels, it will result in severe and permanent consequences such as climate change, loss of plant and animal species, food shortages, and environmental contamination [1,2,3,4,5,6]. The main cause of global warming is the increase in greenhouse gas emissions when people do not stop exploiting and consuming fossil fuels, such as petroleum, natural gas, and coal, which cause the concentration of CO_2_ in the air to increase rapidly. As a result, the CO_2_ levels increased from 316 ppm in 1960 to 417 ppm in 2020, as measured at Mauna Loa Observatory, Hawaii [7]. The global average surface temperature increased by about 0.6 °C over the 20th century [8,9]. The consequences of global warming are not something in the distant future, but are already clearly evident. In response to global warming, the use of environmentally friendly, zero-emission fuels that do not release greenhouse gases is more urgent than ever. Renewable energy sources such as solar, wind, and hydropower are being increasingly utilized to reduce greenhouse gas emissions. However, the intermittent nature of these sources and the lack of effective energy storage systems limit their widespread adoption. Hydrogen, on the other hand, emerged as a promising zero-emission fuel that can be produced from renewable sources and has a high energy density, making it an ideal candidate for energy storage and transportation.

Hydrogen can be produced through several methods, including electrolysis, biomass gasification, and steam methane reforming. Electrolysis, which involves splitting water into hydrogen and oxygen using electricity, is considered the most environmentally friendly method of hydrogen production when powered by renewable energy sources such as wind and solar. The use of renewable energy sources to produce hydrogen can help reduce greenhouse gas emissions and contribute to the transition to a low-carbon economy.

Hydrogen can be used in various applications, including transportation, heating, and power generation. In the transportation sector, hydrogen fuel cell electric vehicles (FCEVs) gained popularity due to their high efficiency, long range, and zero emissions. FCEVs use hydrogen as a fuel to generate electricity, producing only water as a byproduct. In the heating sector, hydrogen can be used in fuel cells to provide combined heat and power for buildings, reducing greenhouse gas emissions from heating. Moreover, hydrogen can be used in industrial processes such as steel and chemical production, where hydrogen can replace fossil fuels as a feedstock, reducing greenhouse gas emissions from these sectors.

Despite the benefits of hydrogen as a zero-emission fuel, its production, storage, and transportation still face challenges. The development of low-cost and efficient hydrogen production methods, safe and efficient hydrogen storage systems, and reliable hydrogen transportation systems are critical to the widespread adoption of hydrogen as a mainstream energy source.

Hydrogen garnered significant attention in recent years as an alternative to fossil fuels, due to its eco-friendliness and abundance. However, the practical application of hydrogen fuel presents a significant challenge. The molecule’s small size (0.289 nm), colorless and odorless nature, and high diffusion coefficient (0.61 cm^2^/s) make it difficult to detect, store, and transport safely. Additionally, its broad flammability range (4–75%) poses a significant safety risk.

Despite these challenges, hydrogen fuel is widely used in various industries, including factories, rockets, automobiles, and metallurgy. However, storing hydrogen gas in high-pressure tanks can lead to significant safety risks due to the potential for explosions caused by even the tiniest leakage.

Therefore, developing a reliable and robust hydrogen gas sensor is crucial to ensure safe storage and transportation of hydrogen fuel. A high-quality sensor must possess high sensitivity and accuracy, a fast response time, and the ability to withstand harsh environments.

To address these challenges, researchers are developing novel hydrogen gas sensors using advanced materials and technology. For instance, metal oxide semiconductors were used as sensing materials due to their high sensitivity to hydrogen gas. Additionally, nanomaterials such as carbon nanotubes, graphene, and metal-organic frameworks were investigated as sensing materials [10,11,12,13,14].

Furthermore, hydrogen gas sensors were developed using various sensing principles, including optical, electrochemical, and thermal. Optical sensors rely on the interaction of hydrogen molecules with light to produce a measurable signal. Electrochemical sensors use an electrode to detect changes in the concentration of hydrogen gas, while thermal sensors measure temperature changes resulting from the catalytic reaction of hydrogen molecules.

Recently, significant progress was made in developing hydrogen gas sensors that can withstand extreme conditions, including high temperatures, pressure, and humidity. These sensors have the potential to revolutionize the safe storage and transportation of hydrogen fuel.

Therefore, developing a reliable and robust hydrogen gas sensor is essential to ensure the safe storage and transportation of hydrogen fuel. Researchers are actively working on developing novel sensing materials and technologies to overcome the challenges associated with hydrogen gas sensing. With continued research and development, hydrogen fuel may soon become a viable alternative to fossil fuels.

In recent decades, semiconductor hydrogen gas sensors based on AlGaN/GaN ] developed rapidly because of their many advantages, such as physical and chemical stability, low cost in mass production, and ability to integrate into the circuit [15,16,17,18,19,20,21,22,23,24]. GaN-based gas sensors especially demonstrated excellent capability for extreme environments such as high temperatures and high radiation [25,26,27,28]. Compared with the AlGaN/GaN diode type, the HEMT-type sensors exhibited several outstanding advantages, including lower theoretical detection limits, the separation of the current-carrying and the sensing mechanism, and the optimization of the sensor’s performance by gate bias modulation [29].

The most crucial component of the sensor is the catalytic structure, which is usually based on metal oxides [30,31,32,33,34] or noble metals [35,36,37,38,39]. Moreover, many studies used the catalyst layer functionalization approach in order to enhance the sensitivity of hydrogen gas sensors. By pulsed-laser irradiation with an optimizing magnitude of the laser power (75 mJ/cm^2^), the NiO thin film exhibited a better response (57.9%) to the hydrogen gas when compared to that of the NiO thin film without irradiation (46.3%) under 3000 ppm of H2 at 175 °C [40]. Other studies developed a p-n metal oxide heterostructure to fabricate high-performance hydrogen gas sensors [41,42]. Additionally, a Pd nanoparticle–decorated SnO_2_ nanotubes sensor, which employed the spillover effect of Pd-nanoparticles and porous microstructure of SnO_2_, showed a huge improvement in hydrogen gas sensing of 4.3 times higher than that of SnO_2_ nanotubes sensor [43]. However, in many cases, the catalyst structure was sensitive to many different gases, leading to the trade-off: the more powerful the catalyst layer, the poorer selectivity, especially for gas sensors using metal oxide catalysts. In order to improve the selectivity of hydrogen gas sensors, the simplest way is to apply a physical filter on the surface of the catalyst material [44,45]. However, to our knowledge, there is no similar study for hydrogen gas sensors based on AlGaN/GaN HEMTs. The reason may lie in the fabrication process, as the AlGaN/GaN HEMTs sensors are fabricated based on photolithography technology. Hence, the application of the thin film on the catalyst layer must be compatible with the fabrication process, which is the major obstacle to the optimization of sensors based on GaN materials.

In this work, we developed a high selectivity hydrogen gas sensor based on AlGaN/GaN HEMTs. A thin layer of WO_3_ was deposited on top of the Pd catalyst layer to achieve high selectivity. The sensor worked in a wide range of temperatures and exhibited the highest sensitivity of 658% towards 4% H_2_ at 200 °C, while the responses to other gases such as NO_2_, CH_4_, CO_2_, NH_3_, and H_2_S were comparably low. These results showed that this sensor is promising for H_2_ detection in a complex gas environment.

## 2. Materials and Methods

**Figure 1:**
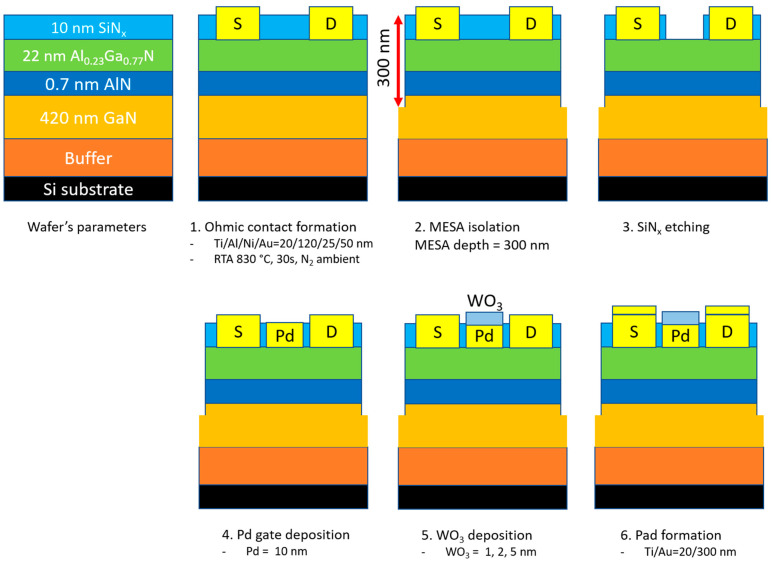
The fabrication process of the Pd-AlGaN/GaN HEMT sensor.

The gas sensors based on AlGaN/GaN HEMTs were fabricated by the conventional photolithography process at the Inter-University Semiconductor Research Center (ISRC), Seoul, Korea. The AlGaN/GaN-on-Si wafer was purchased as a commercial product, which consisted of a 10 nm in situ SiNx layer, a 22 nm Al_0.23_Ga_0.77_N barrier layer, a 0.7 nm AlN nucleation layer, 420 nm i-GaN layer, and buffer layers. First, the source and drain electrodes were patterned by the maskless patterning system, and then, a metal stack of Ti/Al/Ni/Au (20/120/25/50 nm) was deposited by e-beam evaporation. After the lift-off process, the samples were followed by rapid thermal annealing (RTA) at 830 °C for 30 s in N_2_ ambient. Next, the mesa isolation at a depth of 300 nm was patterned and applied by inductively coupled plasma (ICP) etching with a BCl_3_/Cl_2_ mixture to remove the active layers between devices. Then, the gate region was patterned and followed by SiN_x_ etching by ICP etching with SF_6_ plasma. Afterward, a 10 nm Pd layer and a WO_3_ layer were formed by e-beam evaporation and a lift-off process on the gate region. In this work, we fabricated 3 samples with different WO_3_ thicknesses of 1, 2, and 5 nm (labeled S1, S2, and S3, respectively) to investigate the influence of the WO_3_ thickness on hydrogen gas selectivity. Finally, the interconnect bi-layer probing pads of Ti/Au with thicknesses 20/300 nm were formed by e-beam evaporation and lift-off. The dimensions of the gate electrode were 36 µm × 120 µm, the source-gate, and gate-drain spacings were 2 µm. The as-fabricated devices were exposed at 400 °C for 10 min in air for stabilization. The cross-section of the fabrication process is shown in Figure 1.

**Figure 2:**
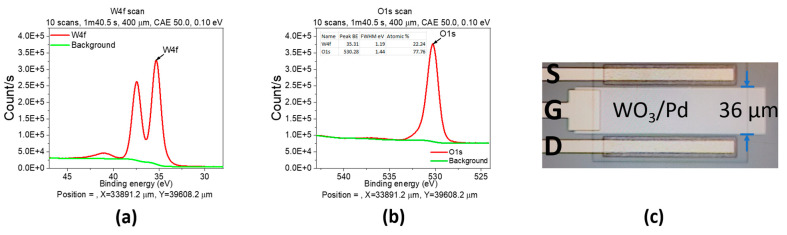
The X-ray photoelectron spectroscopy spectra for W4f and O1s of Si test sample (**a**,**b**) and the microscope image of the fabricated device (**c**).

Thin WO_3_ film was deposited on a test Si wafer using e-beam evaporator from high purity WO_3_ pellets (99.99%) with a Z-ratio of 0.529 [46]. The X-ray photoelectron spectroscopy (XPS) spectra were shown in Figure 2a,b. In Figure 2a, the two main XPS peaks belonged to the typical doublet of W6+ with the binding energy of W (4f_7/2_) centered at 34.9 ± 0.1. On the other hand, the main peak of O (1s) at 530.7 ± 0.1 corresponded to W-O bond (Figure 2b). In addition, the atomic percentage was W:O = 22.24:77.76 (inset of Figure 2b), which is close to the theoretical ratio 25:75, confirming that WO_3_ thin film was successfully deposited.

The microscope image of the fabricated WO_3_/Pd-AlGaN/GaN HEMT device was shown in Figure 2c.

**Figure 3:**
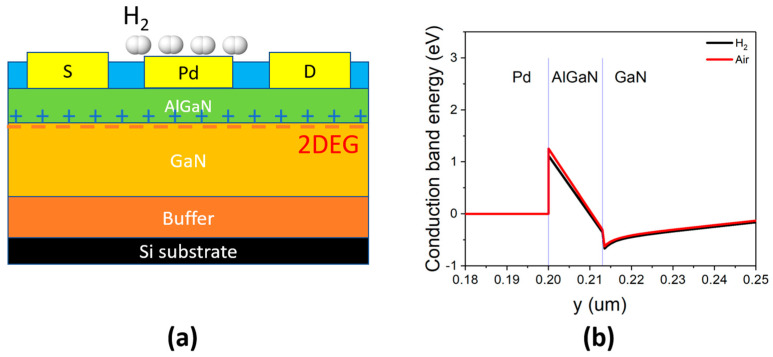
The sensing mechanism of gas sensor based on Pd-AlGaN/GaN HEMT (**a**) and the change of conduction band energy with hydrogen exposure (**b**).

The gas sensors based on Pd-AlGaN/GaN were widely investigated and reported [17,18]. When hydrogen molecules came into contact with the Pd catalyst layer, the Pd layer enabled the hydrogen dissociation into hydrogen ions (H+) on the Pd surface. Due to their tiny size, the positively charged hydrogen ions easily diffused through the gate layer via pores and reach the surface of the AlGaN layer. The presence of the positive ions increased the two-dimensional electron gas (2DEG) concentration and lead to an increase in drain current (Figure 3a). The change of conduction band energy with hydrogen exposure was shown in Figure 3b.

As reported in [47,48], the pure WO_3_ thin film exhibited no reduction reaction to H_2_ at 50–300 °C. Hence, the thin film of WO_3_ acted as a gas filter without compromising Pd’s hydrogen sensing and played an essential role in archiving high selectivity for H_2_ sensing.

**Figure 4:**
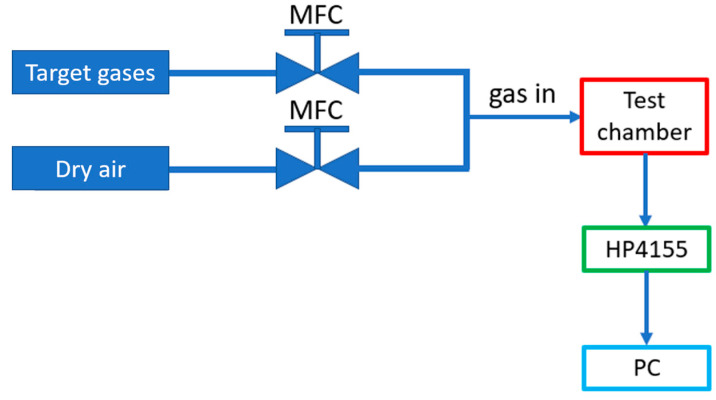
Gas sensor measurement setup.

The gas sensor measurement setup was shown in Figure 4. Gas sources consisted of the target gases (H_2_, NO_2_, CH_4_, CO_2_, NH_3_, and H_2_S) and synthesized dry air (O_2_/N_2_ mixture) as the reference gas. The gas flow was mixed using mass flow controllers (MFCs). The sensors were loaded in a test chamber with a hot chuck to control the operating temperature. Output and gas sensing characteristics were measured by HP 4155A parameter analyzer. The gas flow rate was maintained at 200 sccm in all measurements.

The relative sensitivity of the sensor was defined by the ratio of the drain current change in the target gas to the base current in dry air:

(1) $$Sensitivity\;\left(\%\right)=\frac{\left|I_{0}-I_{gas}\right|}{I_{0}}\times 100=\frac{\left|\Delta I\right|}{I_{0}}\times 100$$

where *I*_0_ and *I_gas_* were the drain current in dry air and target gases, respectively. The response time was defined as the required time for the sensor to reach 90% of the total response from the base current, and the recovery time showed the time required to return to 90% of the base current. The response and recovery times were extracted from the transient characteristics.

## 3. Results

**Figure 5:**
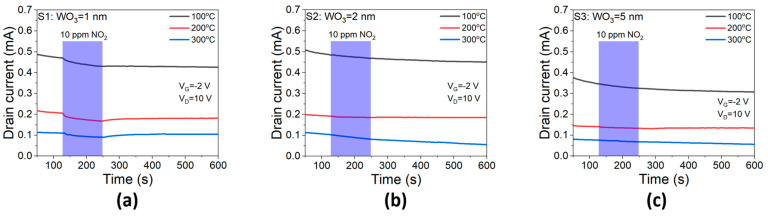
Response to 10 ppm NO_2_ of the sensors at different temperatures. (**a**) 1 nm (**b**) 2 nm (**c**) 5 nm of WO_3_ layer.

Figure 5 showed the drain current response to 10 ppm NO_2_ at 100, 200, and 300 °C. Our previous work [49] reported that high relative sensitivity towards NO_2_ could be achieved in Pd-AlGaN/GaN HEMT sensor by optimizing AlGaN barrier thickness along with gate bias modulation effect. The sensitivity to 10 ppm of NO_2_ at 300 °C was 6% at V_G_ = 0 V and 45.4% at V_G_ = −1 V, which was much improved in the sensor device with 13 nm AlGaN barrier. However, a thin layer of WO_3_ deposited on top of the Pd layer made the sensors remarkably less sensitive to NO_2_ as shown in Figure 5. When Pd gate was covered with 1 nm of WO_3_, the S1 sensor still demonstrated the sensitivity of 8.9%, 18.4%, and 17.2% at 100, 200, and 300 °C, respectively. However, NO_2_ response was diminished significantly in the S2 sensor with 2 nm- WO_3_ layer (Figure 5b), and the S3 sensor with 5 nm WO_3_ lost the NO_2_ sensitivity completely (Figure 5c). The NO_2_ insensitivity indicated that the WO_3_ layer can be utilized as a gas filter against NO_2_. When this filter layer was thick enough beyond 5 nm thickness, the sensor was no longer sensitive to NO_2_. In this work, we found that 5 nm of WO_3_ was enough for NO_2_ insensitivity, while maintaining working stability. Although the WO_3_ thin film was reported to be sensitive to NO_2_ [50,51], our data showed that the WO_3_/Pd dual layer did not have any enhancement for the NO_2_ sensing.

**Figure 6:**
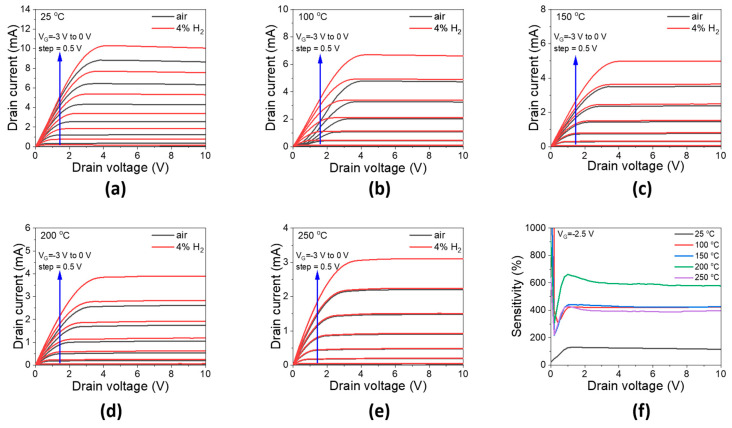
Output characteristics of the S3 sensor in air and 4% H_2_ gas from (**a**) 25, (**b**) 100, (**c**) 150, (**d**) 200, and (**e**) 250 °C at different gate voltages from −3 V to 0 V. (**f**) the sensitivity extracted from output characteristics at gate voltage of −2.5 V.

The WO_3_/Pd-AlGaN/GaN hydrogen gas sensor operated normally in a wide range of temperatures from 25 °C to 250 °C, as shown in Figure 6. At 200 °C, the S3 sensor exhibited the highest relative sensitivity (Figure 6f). These results were similar to other works using Pd-AlGaN/GaN HEMTs sensor. This result indicated that the WO_3_ layer on Pd catalyst did not affect the hydrogen gas sensing mechanism.

**Figure 7:**
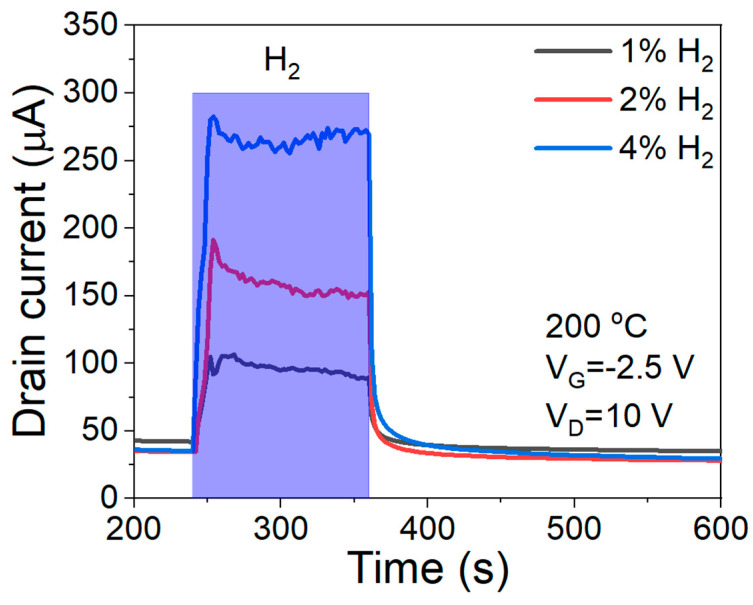
Response to H_2_ gas of S3 sensor at 200 °C.

The thin WO_3_ layer, which acted as a filter or a blocking layer, might degrade the sensor’s performance. However, the WO_3_/Pd-AlGaN/GaN HEMT sensor maintained a high performance for hydrogen sensing, as shown in Figure 7. At 200 °C, the transient characteristics of the sensor showed a fast response (12 s) and recovery times (4 s) while maintaining the high sensitivity of 658% under hydrogen gas of 4% concentration.

**Figure 8:**
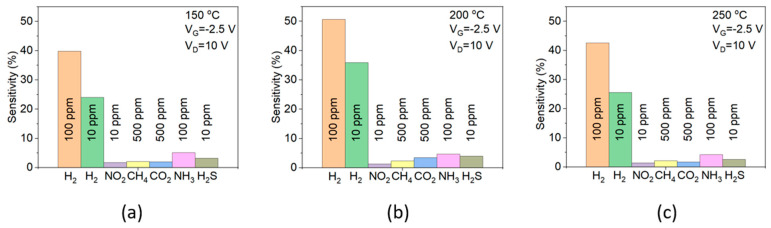
Hydrogen selectivity of S3 sensor at 150 °C (**a**), 200 °C (**b**), and 250 °C (**c**).

The sensitivity of the sensors was measured with the same bias point and temperature under exposure to various gases, including NO_2_, CH_4_, CO_2_, NH_3_, and H_2_S, in order to evaluate the hydrogen selectivity. Unfortunately, the measurement was not conducted in the gas combining condition, due to the limitation of the measurement facility. The sensitivity of our sensors to many different gases was shown in Figure 8. To our knowledge, the hydrogen gas sensor based on AlGaN/GaN with such a high selectivity was rarely reported. The high selectivity of the sensor can be explained by the combination of a high hydrogen-sensitive catalyst and the filter effect of the WO_3_ layer. First, being the smallest gas molecule with a bond length of 0.74 Å [52], hydrogen gas molecules can easily diffuse and penetrate the filter. A similar filter approach was investigated to achieve high hydrogen selectivity. For example, a low porosity of SiO_2_ layer, as a molecular sieve, enhanced the hydrogen selectivity of SnO_2_ thick film [53]. Similarly, SnO_2_ gas sensors with SiO_2_ deposited on the surface showed significant improvement in hydrogen selectivity, whereas no noticeable response toward ethanol, acetone, and benzene were detected [54]. Second, palladium itself is a powerful catalyst with a high affinity toward hydrogen, which allows the effective dissociation of hydrogen molecules into hydrogen atoms and forms the palladium hydrides PdH_x_ [55,56].

**Figure 9:**
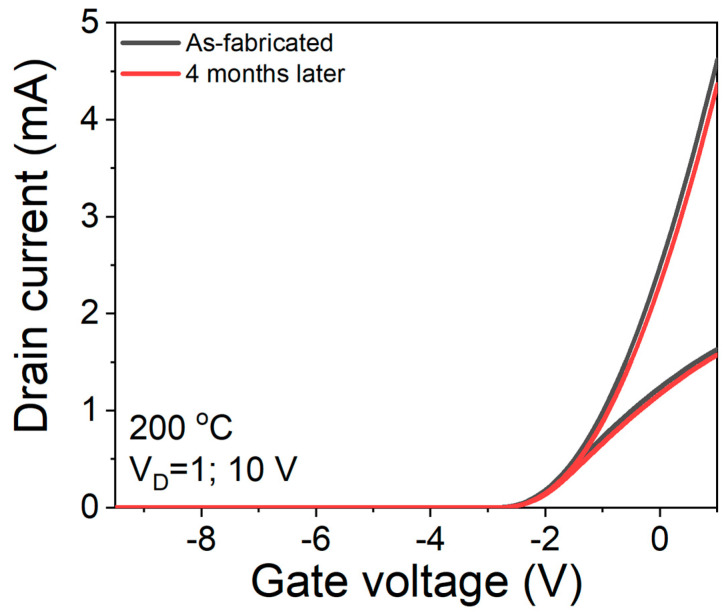
Stability of S3 sensor.

The stability of S3 sensor was exhibited by the transfer characteristics at 200 °C, as shown in Figure 9. After 4 months with various measurements, we observed roughly 7% of the current collapse.

## 4. Conclusions

We reported a high selectivity hydrogen gas sensor based on AlGaN/GaN HEMTs. A thin layer of WO_3_ on top of the Pd layer worked as a gas filter to enhance the sensor selectivity towards hydrogen gas. High selectivity and sensitivity, fast response, and quick recovery times made the sensor ideal for hydrogen gas sensing at a high temperature in a complex gas environment.

## Data Availability

Not applicable.
